# Differential Control of *Yersinia pestis* Biofilm Formation *In Vitro* and in the Flea Vector by Two c-di-GMP Diguanylate Cyclases

**DOI:** 10.1371/journal.pone.0019267

**Published:** 2011-04-29

**Authors:** Yi-Cheng Sun, Alexandra Koumoutsi, Clayton Jarrett, Kevin Lawrence, Frank C. Gherardini, Creg Darby, B. Joseph Hinnebusch

**Affiliations:** 1 Laboratory of Zoonotic Pathogens, Rocky Mountain Laboratories, National Institute of Allergy and Infectious Diseases, National Institutes of Health, Hamilton, Montana, United States of America; 2 Program in Microbial Pathogenesis, Department of Cell and Tissue Biology, University of California San Francisco, San Francisco, California, United States of America; University of Osnabrueck, Germany

## Abstract

*Yersinia pestis* forms a biofilm in the foregut of its flea vector that promotes transmission by flea bite. As in many bacteria, biofilm formation in *Y. pestis* is controlled by intracellular levels of the bacterial second messenger c-di-GMP. Two *Y. pestis* diguanylate cyclase (DGC) enzymes, encoded by *hmsT* and y3730, and one phosphodiesterase (PDE), encoded by *hmsP*, have been shown to control biofilm production *in vitro* via their opposing c-di-GMP synthesis and degradation activities, respectively. In this study, we provide further evidence that *hmsT*, *hmsP*, and y3730 are the only three genes involved in c-di-GMP metabolism in *Y. pestis* and evaluated the two DGCs for their comparative roles in biofilm formation *in vitro* and in the flea vector. As with HmsT, the DGC activity of Y3730 depended on a catalytic GGDEF domain, but the relative contribution of the two enzymes to the biofilm phenotype was influenced strongly by the environmental niche. Deletion of y3730 had a very minor effect on *in vitro* biofilm formation, but resulted in greatly reduced biofilm formation in the flea. In contrast, the predominant effect of *hmsT* was on *in vitro* biofilm formation. DGC activity was also required for the Hms-independent autoaggregation phenotype of *Y. pestis*, but was not required for virulence in a mouse model of bubonic plague. Our results confirm that only one PDE (HmsP) and two DGCs (HmsT and Y3730) control c-di-GMP levels in *Y. pestis*, indicate that *hmsT* and y3730 are regulated post-transcriptionally to differentially control biofilm formation *in vitro* and in the flea vector, and identify a second c-di-GMP-regulated phenotype in *Y. pestis*.

## Introduction

*Yersinia pestis*, the cause of plague, is a Gram-negative bacterium that is transmitted to mammals by infected fleas. It evolved from the enteric pathogen *Yersinia pseudotuberculosis* within the past 20,000 years [1]. During its life cycle, *Y. pestis* colonizes the flea midgut and can form a biofilm in the proventricular valve in the foregut. Growth and consolidation of the biofilm within the proventriculus interferes with or completely blocks normal blood feeding, resulting in regurgitation of bacteria and transmission. Fleas with a blocked proventriculus make prolonged, repeated attempts to feed, enhancing the transmission of the bacteria. Unblocked fleas are also capable of spreading disease by early-phase transmission during the first few days after becoming infected [2], [3], but only blocked or partially blocked fleas can transmit disease after the early phase. Thus, the ability to produce a proventricular biofilm is believed to be crucial for long-term enzootic persistence of *Y. pestis* [3], [4].

When grown at ≤28°C on agar media containing haemin or Congo red (CR), *Y. pestis* adsorbs the dye and forms greenish-brown or red ‘pigmented’ colonies, respectively. The pigmentation (Pgm+) phenotype of *Y. pestis* correlates well, although not perfectly, with biofilm formation [5], [6]. Biofilm formation in the flea and *in vitro* is characterized by a dense aggregate of bacteria embedded within an extracellular matrix (ECM). *Y. pestis* biofilm and Pgm phenotypes require the *hmsHFRS* operon, which is responsible for biosynthesis of the ECM polysaccharide [7]–[9].

As in many other bacteria, ECM production and biofilm development in *Y. pestis* is controlled by bis-(3′-5′)-cyclic dimeric GMP (c-di-GMP), a soluble molecule that functions as a ubiquitous second messenger in bacteria [5], [6], [10]. c-di-GMP stimulates the biosynthesis of adhesin and ECM components and controls the switch between the free-living planktonic and sedentary biofilm-associated lifestyles of many bacteria [11]. c-di-GMP is synthesized by diguanylate cyclase (DGC) enzymes that contain a GGDEF domain and is hydrolyzed by phosphodiesterase (PDE) enzymes that contain an EAL or HD-GYP domain [12]–[14]. Bioinformatic analyses indicate that GGDEF and EAL/HD-GYP domain genes are often highly abundant in bacterial genomes [15]. This redundancy suggests that intracellular levels of c-di-GMP may reflect the cumulative activities of a complex composite of different GGDEF and EAL/HD-GYP family member pairs.

In this study, we evaluated the role of the ten known or putative DGC- and PDE-encoding genes of *Y. pestis* in regulation of the biofilm phenotype, and show that the two DGCs of *Y. pestis* make different, environment-dependent contributions to biofilm formation. The DGC encoded by the *Y. pestis hmsT* gene is sufficient for normal *in vitro* biofilm formation, whereas the DGC encoded by the y3730 gene has very little effect on *in vitro* biofilm formation but has the major role in producing proventricular-blocking biofilm in the flea. We also identify autoaggregation as a second c-di-GMP-controlled phenotype in *Y. pestis* that is unrelated to biofilm exopolysaccharide production.

## Results

### Effect of *Y. pestis* GGDEF-, EAL-, and HD-GYP- domain genes on *in vitro* pigmentation and biofilm phenotypes

**Figure 1:**
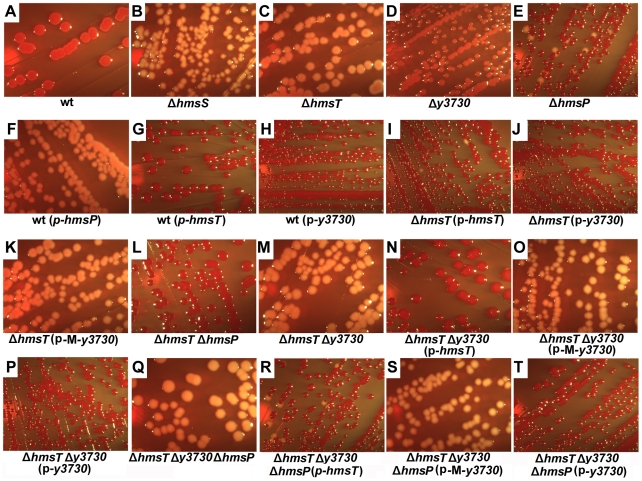
Pigmentation phenotype of *Y. pestis* strains on LB-Congo red agar. See text and Table 3 for description of the strains.

The *Y. pestis* KIM strain contains ten genes that are predicted to encode GGDEF-domain (Pfam family PF00990), EAL-domain (Pfam family PF00563), or HD-GYP-domain (Pfam family PF0196) proteins (Table 1) [15]–[17]. To determine which of these could potentially participate in proventricular biofilm formation in the flea, we began by evaluating the effect each of these genes had on *in vitro* biofilm formation. Each gene was individually deleted from *Y. pestis* KIM6+. The resulting series of mutant strains was first tested for pigmentation phenotype using a formulation of Congo red agar better able to detect relative increases and decreases in pigmentation than the classic Surgalla Congo red agar plates [18]. The plates were incubated at 28°C, a temperature at which the KIM6+ wild-type strain exhibits an intermediate level of pigmentation (Fig. 1A). A known nonpigmented mutant deleted of *hmsS* was used as a negative control (Fig. 1B). As expected, the *hmsT* mutant formed nonpigmented colonies and the *hmsP* mutant formed hyperpigmented colonies that were a much darker red than wild-type colonies (Fig. 1 A, C, E). Deletion of the GGDEF domain gene y3730 resulted in a very subtle decrease in pigmentation (Fig. 1D), but deletion of the other seven genes of interest did not affect the CR phenotype (data not shown).

**Table 1:** Known and putative DGC and PDE proteins in *Y. pestis* KIM6+.

Predicted function	Gene No.^a^	Gene name	Motif	*Y. pstb* b homologue	Comparison with *Y. pstb* homologue
DGC	y2559	-	GGDEF	YPTB1628	65% identity^c^
	y3730	*-*	GGDEF	YPTB0592	100% identity
	y3756	*hmsT*	GGDEF	YPTB0570	98% identity
PDE	y1612	-	ELL	YPTB2605	99% identity
	y2909	*rtn*	EAL	YPTB1308	93% identity^c^
	y3841	-	ELL	YPTB3828	98% identity
	y2472	-	HI-GYP	YPTB1709	30% identity^c^
DGC and/or PDE	y3389	-	GGDEF and EAL	YPTB3308	92% identity^c^
	y3832	*hmsP*	SKTEF and EAL	YPTB3836	99% identity
Other	y0203	*csrD*	LNSDI and EII	YPTB3566	99% identity

a *Y. pestis* KIM annotation number.

b *Y. pseuodotuberculosis* IP32953 annotation number.

c Difference due to N-terminal truncation of the predicted *Y. pestis* protein.

**Figure 2:**
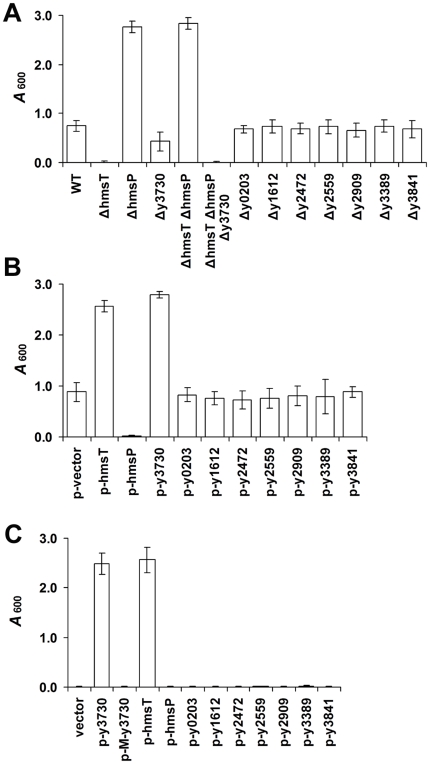
Effect of *Y. pestis* GGDEF-, EAL-, and HD-GYP-domain genes on *in vitro* biofilm formation. A, B. Relative amounts of adherent biofilm made by *Y. pestis* KIM6+ parent strain and isogenic derivatives deleted of (A) or overexpressing (B) one of the genes listed in Table 1. C. Quantitation of biofilm made by the *hmsT hmsP* y3730 triple mutant strain overexpressing one of the genes listed in Table 1, or the mutated y3730 GGAAF allele. The mean and standard deviation of two or more independent experiments are indicated.

The mutants were further tested for their ability to form biofilm in microtiter plates at room temperature using a crystal violet staining assay (Fig. 2A). Consistent with previous results [6], deletion of *hmsT* abolished biofilm formation whereas deletion of *hmsP* resulted in increased biofilm formation. In addition, deletion of y3730 resulted in slightly reduced (not statistically significant) biofilm formation, but deletion of the other seven genes did not affect the ability to produce biofilm in this assay (Fig. 2A).

To further assess their function, the genes encoding DGC and PDE domains were overexpressed in *Y. pestis* KIM6+ from a high-copy plasmid vector. As expected, the strain that overexpressed *hmsP* formed white colonies on the CR plates and the strain that overexpressed *hmsT* formed hyperpigmented colonies (Fig. 1F, G). The strain that overexpressed y3730 also formed hyperpigmented colonies (Fig. 1H). However, overexpression of any of the other seven genes in *Y. pestis* KIM6+ did not affect the CR phenotype (data not shown). Complementary results were observed with the microtiter plate biofilm assay, in which increased biofilm was produced only in strains overexpressing *hmsT* or y3730 (Fig. 2B).

**Figure 3:**
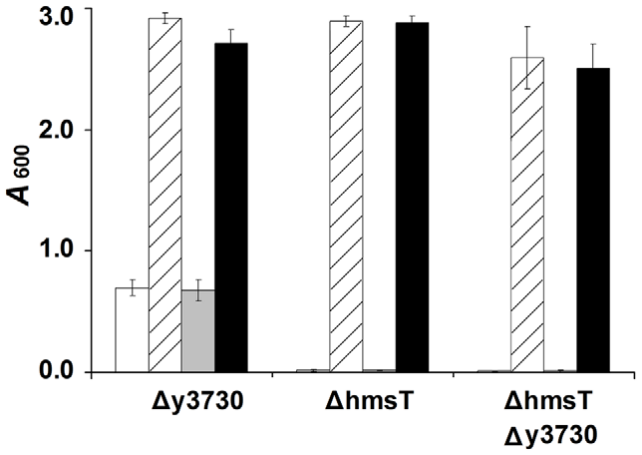
Relative amount of *in vitro* biofilm made by the *Y. pestis* y3730, *hmsT*, and y3730 *hmsT* mutant strains after transformation with the empty plasmid vector, (white bars) or the plasmid containing wild-type y3730 (hatched bars), mutated y3730 (grey bars), or *hmsT* (black bars). The mean and standard deviation of two or more independent experiments are indicated.

To further confirm that y3730, *hmsT*, and *hmsP* are the only c-di-GMP-metabolizing genes involved in biofilm formation, we constructed *hmsT* y3730 double mutant, *hmsT hmsP* double mutant, and *hmsT hmsP* y3730 triple mutant strains of *Y. pestis*. As predicted, the *hmsT hmsP* double mutant still formed red colonies on CR plates (Fig. 1L) and strong biofilm *in vitro* (Fig. 2A), whereas the triple deletion mutant formed white colonies on the CR plate (Fig. 1Q) and no biofilm *in vitro* (Fig. 2A). Complementation of the *hmsT*, *hmsT* y3730 and *hmsT hmsP* y3730 mutants with either *hmsT* or y3730 resulted in an equivalent increase in biofilm formation (Fig. 1I-T; Fig. 2C; Fig. 3). However, a plasmid containing a mutated y3730 allele (M- y3730) in which the GGDEF-encoding domain was changed to a GGAAF-encoding domain failed to complement y3730 or *hmsT* mutation (Fig. 1K,O; Fig. 2C; Fig. 3). Transformation of the triple mutant with high-copy number plasmids containing any of the other eight genes encoding putative DGC and PDE proteins (Table 1) did not change the biofilm-negative and nonpigmented phenotype of the mutant (Fig. 2C and data not shown).

Finally, we assayed intracellular levels of c-di-GMP in the *Y. pestis hmsT* y3730 *hmsP* triple mutant before and after it was transformed with the p-y3730 plasmid. As predicted, no c-di-GMP was detected in cell lysates of the triple mutant. Synthesis of c-di-GMP was observed when the mutant was transformed with plasmids containing y3730 or *hmsT*, but not with M-y3730 (Fig. S1).

### Differential effect of y3730 and *hmsT* mutation on *Y. pestis* biofilm formation in fleas

The preceding results indicated that only the GGDEF domain genes y3730 and *hmsT* have DGC function that potentially induces proventricular biofilm formation. To investigate the role of y3730 during infection of the flea vector, the *Y. pestis* KIM6+ parent strain and the y3730 and *hmsT* mutants were tested for their ability to infect and produce biofilm-dependent proventricular blockage in fleas (Table 2). Infection with *Y. pestis* KIM6+ resulted in blockage of 38 to 45% of the fleas, consistent with previous results [7]. Although the *Y. pestis hmsT* mutant forms nonpigmented colonies (Fig. 1C) and little or no biofilm *in vitro* (Fig. 1C; Fig. 2A, C; Fig. 3), it was still able to block 16 to 20% of infected fleas, a reduction in blockage of about 50% (*P*<0.0001 by Fisher's exact test). In contrast, although deletion of y3730 had little effect on *in vitro* pigmentation or biofilm-forming ability (Fig. 1D, Fig. 2A, Fig. 3), proventricular blockage of fleas infected with the y3730 mutant was only 1 to 5%, a rate significantly lower than that of fleas infected with KIM6+ wild-type or *hmsT* mutant (*P*<0.0001). The blockage rates produced by the *Y. pestis* y3730 single mutant and the *hmsT* y3730 double mutant were not significantly different (*P* = 0.16), further indicating that y3730 has the predominant role in biofilm formation in the flea. The severe defect in biofilm-dependent proventricular blockage of the y3730 mutant could be restored by complementation with either y3730 or *hmsT* on a high-copy number plasmid; however, overexpression of the M-y3730 allele lacking the GGDEF domain did not complement the y3730 mutation (Table 2).

**Table 2:** Infection and blockage of fleas by *Y. pestis* strains.

Strain	*Y. pestis* CFU/flea^a^ at:		Fleas infected at 28 days	Blockage rate	Fleas(n)
	0 day	28 days			
KIM6+ wild type	8.2×10^4^±5.5×10^4^1.8×10^5^±1.3×10^5^	4.8×10^5^±2.4×10^5^6.0×10^5^±3.4×10^5^	100%65%	38%45%	106113
KIM6+ Δ*hmsT*	5.1×10^4^±2.8×10^4^9.2×10^4^±4.9×10^4^	3.1×10^5^±1.3×10^5^6.3×10^5^±3.3×10^5^	90%80%	20%16%	106119
KIM6+ Δ*hmsT* (p-*hmsT*)	1.0×10^5^±7.7×10^4^1.2×10^5^±1.4×10^5^	7.7×10^5^±4.5×10^5^5.8×10^5^±4.2×10^5^	95%90%	46%31%	10590
KIM6+ Δy3730	5.8×10^4^±7.3×10^4^4.6×10^4^±4.1×10^4^	2.9×10^5^±3.1×10^5^3.2×10^5^±3.4×10^5^	70%70%	1%5%	88108
KIM6+ Δy3730 (p-y3730)	4.2×10^4^±4.0×10^4^1.5×10^5^±0.8×10^5^	6.4×10^5^±3.7×10^5^7.2×10^5^±2.4×10^5^	50%100%	37%47%	106106
KIM6+ Δy3730 (p-M-y3730)	3.3×10^5^±2.2×10^5^4.6×10^4^±2.0×10^4^	4.4×10^5^±3.2×10^5^4.3×10^5^±2.7×10^5^	95%95%	8%6%	108107
KIM6+ Δy3730 (p-*hmsT*)	2.7×10^5^±1.8×10^5^1.5×10^5^±0.8×10^5^	8.8×10^5^±2.2×10^5^8.5×10^5^±3.0×10^5^	95%90%	42%36%	101110
KIM6+ Δ*hmsT*Δy3730	1.6×10^5^±1.5×10^5^2.0×10^4^±1.9×10^4^	2.7×10^5^±3.9×10^5^3.6×10^4^±4.4×10^4^	90%24%	0%2%	107109
KIM6+ Δ*hmsT*Δy3730 (p-y3730)	6.2×10^4^±5.9×10^4^1.7×10^4^±1.2×10^4^	5.0×10^5^±3.6×10^5^5.4×10^5^±3.0×10^5^	70%85%	24%33%	108111

The results of two experiments with each bacterial strain are shown.

a Mean±SD.

The infection rate and the mean bacterial load (CFU per flea) at 4 weeks (Table 2) were significantly lower in fleas infected with the *Y. pestis hmsT* y3730 double mutant compared to fleas infected with wild-type KIM6+ (*P*<0.05), consistent with previous reports that lack of ability to form biofilm correlates with decreased persistence in the flea [7], [19]. The infection rate and bacterial load in fleas infected with the y3730 mutant was not significantly different than in fleas infected with wild-type or *hmsT* mutant *Y. pestis* (Table 2); therefore the highly significant difference in biofilm-dependent blockage produced by the y3730 mutant cannot be accounted for by any decreased ability to produce a chronic infection in the flea gut.

### Transcription of y3730 and *hmsT* is upregulated in the flea

**Figure 4:**
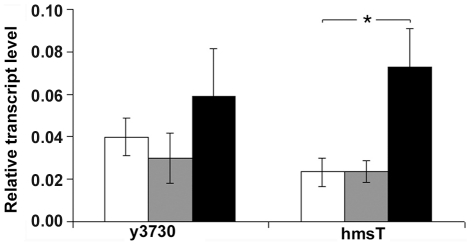
Expression of *Y. pestis* y3730 and *hmsT in vitro* and in the flea. Relative amounts of y3730 and *hmsT* mRNA expressed in fleas (black bars), *in vitro* biofilm cultures (grey bars) and *in vitro* planktonic cultures (white bars) are shown. The mean and SD of three independent experiments is indicated. *P<0.05 by t-test.

Control of DGC and PDE expression at the transcriptional level in different environments can be important for c-di-GMP regulation in bacteria [10]. We compared transcript levels of *hmsT* and y3730 in *Y. pestis* cells isolated from fleas, *in vitro* biofilms, and planktonic cultures. The expression pattern of both genes was similar in the three different growth conditions, with highest expression detected in the flea (Fig. 4). The relative transcript level of *hmsT* was even higher than that of y3730 in the flea, but this difference was not statistically significant. Thus, differential transcriptional regulation of *hmsT* and y3730 does not appear to account for the predominant role of *hmsT* during *in vitro* growth or the predominant role of y3730 in the flea.

### DGC activity is required for the autoaggregation phenotype of *Y. pestis*

**Figure 5:**
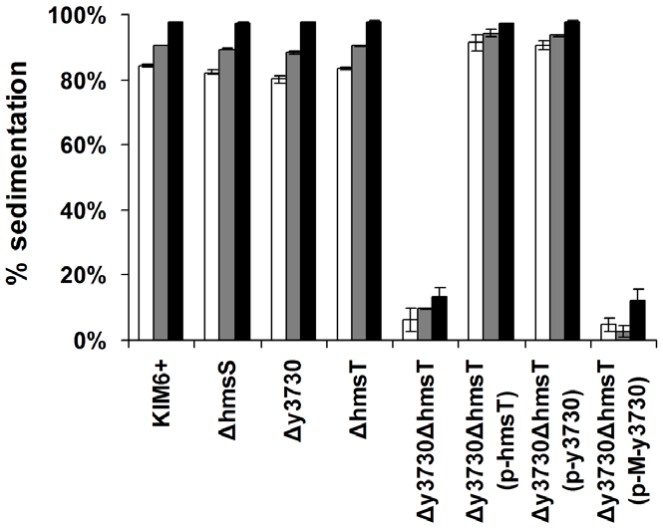
y3730 and *hmsT* are required for *Y. pestis* autoaggregation. The % sedimentation of bacterial growth in liquid cultures was determined by spectrophotometry after 1, 2, and 12 hours of stasis (white, grey, and black bars, respectively). The mean and SD of two independent experiments performed in duplicate are indicated.

*Y. pestis* autoaggregates during growth in liquid culture, and this phenotype is not dependent on Hms-dependent ECM or biofilm [20], [21]. In a sedimentation assay to quantitate relative autoaggregation, the *Y. pestis hmsS* mutant aggregated in culture as rapidly as wild type *Y. pestis* KIM6+ (Fig. 5), verifying that Hms-dependent ECM is not required for the phenotype. To our surprise, however, disruption of both *hmsT* and y3730, but not either one alone, resulted in loss of autoaggregation in *Y. pestis* (Fig. 5). The autoaggregation phenotype of the double mutant could be restored by complementation with either y3730 or *hmsT*, but not M-y3730. These results suggest that c-di-GMP is involved in the autoaggregation of *Y. pestis* in an ECM-independent manner.

Cyclic-di-GMP signaling pathways are known to control the expression of virulence factors, and autoaggregation is reported to be important for virulence in some bacteria [22]–[24]. Therefore, we also tested the effect of complete loss of *Y. pestis* c-di-GMP synthetic ability on virulence in a murine model of plague. No significant difference in mortality or time to disease onset was observed in mice injected with the virulent *Y. pestis* KIM6+ (pCD1-kan) strain or with an isogenic *hmsT* y3730 (pCD1-kan) double mutant, indicating that loss of DGC activity does not decrease the virulence of *Y. pestis* for mice (Fig. S2).

## Discussion

The biofilm phenotype is important in maintaining flea to mammal transmission cycles of plague, because growth as a biofilm enhances *Y. pestis* colonization of the flea and transmission by fleabite. *Y. pestis* also grows as a biofilm *in vitro* in a temperature-dependent fashion. At temperatures at or below 28°C (corresponding to the ambient temperatures experienced in the flea vector), the ECM required for the pigmentation and biofilm phenotypes is synthesized by the *hsmHFRS* genes, but at 37°C (corresponding to the mammalian host body temperature), ECM and biofilm are not produced. To date, two mechanisms have been identified that control biofilm development in *Y. pestis*. As in many bacteria, the second messenger c-di-GMP promotes biofilm development by activating the synthesis of the ECM. Additionally, posttranscriptional regulation by proteolysis of HmsH, HmsR, and HmsT at 37°C is responsible for the lack of biofilm development at high growth temperatures [25].

When we initiated our study, only two enzymes involved in c-di-GMP metabolism, HmsT, a GGDEF-domain diguanylate cyclase, and HmsP, a EAL-domain phosphodiesterase had been characterized in *Y. pestis* [6], [25]–[27]. A very recent study has identified y3730 as a second DGC gene, also as part of a systematic evaluation of all ten GGDEF-, EAL-, and HD/GYP domain-encoding genes of *Y. pestis* [28]. This study also demonstrated that HmsT, HmsP, and Y3730 were the only functional c-di-GMP-metabolizing enzymes in *Y. pestis*, but that y3730 had little effect on the *in vitro* biofilm phenotype [28]. We have independently confirmed these results, using a somewhat different strategy. We systematically i) deleted and ii) overexpressed all *Y. pestis* genes that were predicted to encode proteins potentially involved in the metabolism of c-di-GMP and examined the effect on biofilm-related phenotypes. Consistent with the recent study, we also identified y3730 as the only other *Y. pestis* gene besides *hmsT* and *hmsP* that is required for pigmentation and biofilm phenotypes and provide confirmatory data that y3730, like *hmsT*, encodes a functional GGDEF-domain DGC that synthesizes c-di-GMP.

A striking difference was seen in the relative roles of the two *Y. pestis* DGCs on biofilm formation *in vitro* and in the flea. Deletion of *hmsT* alone virtually eliminated *in vitro* pigmentation and biofilm phenotypes (Fig. 1C, Fig. 2A, C; Fig. 3), consistent with previous reports [6], [25], [27]. Deletion of *y3730*, however, resulted in at most a very subtle decrease in *in vitro* pigmentation and biofilm (Fig. 1D, Fig. 2A), which probably is why y3730 was not discovered in previous random mutagenesis - *in vitro* screening strategies [27]. In contrast, y3730 deletion had the major effect on *in vivo* biofilm formation in the flea (Table 2). Differential regulation of *hmsT* and y3730 transcription does not appear to account for the different *in vitro* and *in vivo* phenotypes, because the two genes were transcribed equivalently in both environments, with the expression of both being upregulated in the flea (Fig. 4). One common means by which the enzymatic activity of GGDEF and EAL domain proteins is differentially modulated is via the presence or absence of additional signal input domains [10]. Interestingly, y3730, but not *hmsT*, encodes a HAMP signaling domain in addition to GGDEF, signal peptide and two widely-spaced transmembrane domains. This pattern is typical of tripartite proteins in which a periplasmic input region responds to an environmental signal that is transduced via the HAMP signal converter to activate a cytoplasmic output domain, in this case GGDEF [29], [30]. Thus, Y3730 may use c-di-GMP as a second messenger to link a specific environmental signal detected only in the flea to the appropriate physiological response– development of a biofilm. If so, the inducing environmental signal presumably is not present or much weaker *in vitro*, where phenotypes dependent on the DGC activity of Y3730 are not detected unless y3730 is highly overexpressed or *hmsP* is deleted. This is consistent with the finding that HmsT is responsible for 75–80% of the intracellular c-di-GMP synthesized by culture-grown *Y. pestis* [28]. Differential stability, localization, or protein-protein interactions of Y3730 and HmsT in the two environments could also contribute to differential activity of the two DGCs *in vitro* and in the flea. For example, direct interaction among HmsT, HmsP, and other Hms proteins in the inner membrane of *Y. pestis* has been demonstrated to be important for regulation of biofilm production *in vitro* [31].

Seven other *Y. pestis* genes predicted to encode GGDEF-, EAL-, or HD-GYP- domain proteins were also analyzed, but none of them had any effect on pigmentation or biofilm phenotypes (Fig. 2). One of them, y0203, contains degenerate GGDEF and EAL domains and is an ortholog of *csrD*, which is not involved in c-di-GMP metabolism but controls the degradation of CsrB/CsrC RNAs in *E. coli* [32]. y1612 and y3841 encode an ELL rather than an EAL domain, and the other four have an N-terminal truncation compared to their *Y. pseudotuberculosis* orthologs (Table 1). Of these, y2909, y3389, and y2559 have been shown to be pseudogenes in *Y. pestis* [28].

In summary, our results confirm that only three c-di-GMP metabolizing proteins– two DGCs (HmsT and Y3730) and one PDE (HmsP) are involved in regulation of biofilm formation in *Y. pestis*, and demonstrate that the role of Y3730 in biofilm production is much greater in the flea than in culture media. We also discovered a second c-di-GMP-dependent phenotype in *Y. pestis*– autoaggregation. This phenotype is independent of c-di-GMP control of ECM production and may reflect the regulation of non-ECM surface components involved in the biofilm lifestyle. Although c-di-GMP levels are known to regulate the expression of virulence factors and pathogenesis in other bacteria, loss of the two DGCs did not significantly affect pathogenesis in a mouse model of bubonic plague (Fig. S2), suggesting that c-di-GMP does not control the expression of essential virulence factors in *Y. pestis*. This is consistent with Bobrov *et al.* [28], who reported that loss of DGC activity did not affect *Y. pestis* virulence in mouse models of both bubonic and pneumonic plague; and that increased c-di-GMP levels resulted in decreased virulence.

*Y. pestis* evolved from *Y. pseudotuberculosis* within the last 20,000 years, while *Y. pseudotuberculosis* and *Y. enterocolitica* diverged about 200 million years ago [1], [33], [34]. *Y. enterocolitica* has 25 genes encoding proteins with GGDEF, EAL, or HD-GYP domains [33], whereas *Y. pseudotuberculosis* has only ten [34]. *Y. enterocolitica* and *Y. pseudotuberculosis* are both food- and water-borne enteric pathogens with similar lifestyles, so it is an interesting question as to why so many DGC and PDE enzymes have been lost in *Y. pseudotuberculosis*. A complex c-di-GMP signaling pathway network may be beneficial for survival in the environment. For example, *Y. pseudotuberculosis* can form biofilm *in vitro* and on the surface of *Caenorhabditis elegans* nematodes, but not in fleas [35]–[37]. In addition, *Y. pseudotuberculosis*, unlike *Y. pestis*, is motile in some environmental conditions, a phenotype that is also often controlled by c-di-GMP. With only three remaining functional DGC and PDE genes, *Y. pestis* appears to have simplified c-di-GMP signaling pathways even further, with the primary role of enhancing transmissibility by controlling biofilm formation in the flea. It is possible that loss of function of some DGCs and PDEs present in the *Y. pseudotuberculosis* progenitor might have favored biofilm formation in the flea, and thus mutational loss of these genes may have been positively selected during the evolution of *Y. pestis*.

## Materials and Methods

### Bacterial strains and plasmids

The strains and plasmids used are shown in Table 3. The *Y. pestis* KIM6+ strain was used in this study [38]. A series of mutant KIM6+ strains in which one of each of the ten GGDEF, EAL, or HD-GYP-encoding genes listed in Table 1 was individually deleted and replaced with a chloramphenicol (cat) or kanamycin (kan) resistance gene cassette was made using the λ Red recombination method [39]–[41]. The double *hmsT* y3730 and triple *hmsT* y3730 *hmsP* mutant strains were made similarly by consecutive replacement of the y3730 and *hmsP* genes with kan and tetracycline resistance genes, respectively, in the Δ*hmsT*::*cat* mutant strain.

**Table 3:** Strains and plasmids used in this study.

Strain or plasmid	Genotype and/or description	Reference or source
*Y. pestis* KIM6+ strains:		
KIM6+	wild type	[38]
Δ*hmsS*	Δ*hmsS::cat*	[40]
Δ*hmsT*	Δ*hmsT::cat*	This study
Δy3730	Δy3730::*kan*	This study
Δ*hmsP*	Δ*hmsP::kan*	This study
Δy0203	Δy0203::*kan*	This study
Δy1612	Δy161*2::kan*	This study
Δy2472	Δy2472::*kan*	This study
Δy2559	Δy255*9::kan*	This study
Δy2909	Δy2909::*kan*	This study
Δy3389	Δy3389::*kan*	This study
Δy3841	Δy3841::*kan*	This study
Δ*hmsT* Δy3730	Δ*hmsT::cat* Δy3730::*kan*	This study
Δ*hmsT* Δ*hmsP*	Δ*hmsT::cat* Δ*hmsP::kan*	This study
Δ*hmsT* Δy3730 Δ*hmsP*	Δ*hmsT::cat* Δy3730*::kan* Δ*hmsP::tet*	This study
KIM6+ (pCD1-kan)	KIM6+ with pCD1-kan	This study
KIM6+ Δ*hmsT* Δy3730 (pCD1-kan)	KIM6+ Δ*hmsT::cat* Δy3730::*kan* with pCD1-kan	This study
Plasmids:		
p-*hmsT* (pCBD26)	*hmsT* in pCR2.1-TOPO	[41]
p-y3730	y3730 in pUC18	This study
p-*hmsP*	*hmsP* in pUC18	This study
p-y0203	y0203 in pCR2.1-TOPO	This study
p-y1612	y1612 in pCR2.1-TOPO	This study
p-y2472	y2472 in pUC18	This study
p-y2559	y2559 in pCR2.1-TOPO	This study
p-y2909	y2909 in pCR2.1-TOPO	This study
p-y3389	y3389 in pCR2.1-TOPO	This study
p-y3841	y3841 in pCR2.1-TOPO	This study
p-M-y3730	y3730 with mutated GGDEF domain (D331A,E332A) in pUC18	This study
pCD1-kan	pCD1 virulence plasmid *with kan* cassette inserted into *yadA*	This study

*Y. pestis* strains overexpressing one of each of the ten GGDEF/EAL/HD-GYP genes (Table 1) were made by first cloning a wild-type copy of each gene, PCR-amplified from KIM6+ using specific primers, into the high-copy number plasmids pUC18 or pCR2.1-TOPO (Invitrogen). These plasmids were then individually transformed into the KIM6+ parent strain by electroporation. A mutated version of the y3730 gene in which the GGDEF-encoding domain was replaced by a GGAAF-encoding domain (resulting in Y3730 D331A, E332A) was prepared by site-specific mutagenesis of the pUC18::y3730 plasmid (p-y3730) using mutagenic primers [42]. The resulting plasmid (p-M-y3730) was used to transform KIM6+. For virulence tests, the KIM6+ and Δy3730 Δ*hmsT* strains were transformed with the pCD1 virulence plasmid from *Y. pestis* KIM5 [43] containing a kan gene inserted into the *yadA* pseudogene. Oligonucleotide primers used for construction of the strains and plasmids are listed in supplemental Table S1. All strains were verified by PCR, DNA sequencing, or plasmid complementation, as appropriate.

### Congo red (CR) pigmentation phenotype assays

The strains were streaked on LB agar (1% tryptone, 0.5% yeast extract, 0.5% NaCl, 1.5% agar) supplemented with 0.01% Congo red and colonies were observed visually for pigmentation phenotype (adsorption of the Congo red dye) after growth for two days at 28°C.

### Microtiter plate biofilm assays

Bacteria were grown in LB broth supplemented with 4 mM CaCl_2_ and 4 mM MgCl_2_ for 24 h at room temperature and diluted to *A*_600_ 0.02 in the same medium. 100 µl aliquots were added to wells of 96-well polystyrene dishes, which were incubated with shaking at 250 rpm for 24 h at room temperature. Media and planktonic cells were removed, the wells were washed four times with water, and the adherent biofilm was stained with 200 µl of 0.01% crystal violet for 15 min. The wells were washed four times with water, bound dye was solubilized with 200 µl of 80% ethanol-20% acetone, and the *A*_600_ was measured. Background absorbance for uninoculated control wells was subtracted. The mean and SD was calculated from three independent experiments with at least three replicates.

### Flea infections

*Xenopsylla cheopis* fleas were fed a single infectious blood meal containing ∼5×10^8^ *Y. pestis* CFU/ml using an artificial feeding system [7], [44]. A sample of 20 female fleas was collected immediately after the infectious blood meal, placed at −80°C, and subsequently used for CFU plate count determinations of the average infectious dose per flea. An additional group of 88 to 119 fleas (approximately equal numbers of males and females) that took an infectious blood meal was maintained at 21°C for four weeks, during which time the fleas were fed twice-weekly on uninfected mice. Immediately after each of these feedings, all fleas were individually examined under a dissecting microscope to determine how many were blocked by proventricular biofilm [7]. After 4 weeks, a sample of 20 surviving female fleas was collected to determine the infection rate and average CFU per infected flea by plate count. Two independent infection experiments were done with each strain. Flea blockage rate and the infection rate at 4 weeks after infection with the different *Y. pestis* strains were analyzed by two-tailed Fisher's exact test, and differences in the mean CFU per flea at 4 weeks were analyzed by one-way ANOVA with Dunnett's post-test, using GraphPad Prism software.

### Quantitative real time PCR

Total RNA of *Y. pestis* KIM6+ was isolated from cells isolated from *in vitro* stationary phase planktonic cultures, *in vitro* biofilms, and infected fleas by using the RNeasy Mini Kit (Qiagen). For the *in vitro* samples, overnight *Y. pestis* cultures were diluted to *A*_600_ 0.02 in 50 ml LB broth supplemented with 4 mM CalCl_2_ and 4 mM MgCl_2_, and grown 24 h with shaking in 250 ml bottles at room temperature. Planktonic cells in the supernatant and biofilm growth adherent to the walls of the culture vessel were separately collected for RNA purification. *Y. pestis* was recovered from pooled, dissected midguts of fleas two weeks after infection. Residual DNA was removed from the RNA samples by treatment with rDnase I (Ambion) and absence of DNA was confirmed by PCR. cDNA was synthesized from the RNA and used for quantitative PCR on an ABI Prism 7900 sequence detection system (Taqman, Applied Biosytems). The reaction contained oligonucleotide primers, probes and the Taqman universal PCR master mix (Applied Biosystems). The quantity of mRNA for each experimental gene was normalized relative to the quantity of the reference gene *crr* (y1485), whose expression level is not affected by in *vivo* or *in vitro* growth conditions [45]. The ratio of the normalized mRNA quantity of y3730 and *hmsT* to *crr* was calculated from three independent assays. Primers and probe sets used are listed in supplemental material Table S1.

### Detection of c-di-GMP

c-di-GMP was detected using high-performance liquid chromatography (HPLC) as described previously [46], [47]. Overnight cultures of the *Y. pestis* Δ*hmsT*Δy3730 Δ*hmsP* strain transformed with different recombinant plasmids or with the empty plasmid vector were diluted 1∶30 into 40 ml of LB medium supplemented with 100 mg/l carbenicillin and grown at 28°C for 8 to10 h with shaking at 250 rpm. Formaldehyde (final concentration of 0.18%) was added to cultures and cells were harvested by centrifugation at 3,000 g for 10 min at 4°C. The pellets were washed with 10 mL of phosphate buffered saline (pH 7) with 0.18% formaldehyde and recentrifuged. The bacterial pellets were resuspended in 1 ml of deionized water and heated at 99°C for 15 min. 2 ml of 95% ethanol were added and the lysate was centrifuged. Supernatants were reextracted with 2 ml of 70% ethanol. Pooled supernatants were evaporated, and pellets were dissolved in 100 µl of 0.15 M triethyl ammonium acetate (TEAA, pH 5.0) and centrifuged 15 min at 16000× g. 20 µL of supernatant was fractionated using an Agilent 1200 Series HPLC (Agilent) with a reverse-phase C18 column (Suplecosil LC-18T, 250×4.6 mm, 5 µm; Supelco). Separations were conducted in 0.15 M TEAA at a 1 mL/min flow rate from 20 µl sample injections, using gradient elution with 0 to 15% acetonitrile with detection at 254 nm. Synthetic c-di-GMP (BIOLOG Life Science Institute) was dissolved in 0.15 M TEAA and used as a standard for peak identification and quantification.

### Autoaggregation assays

*Y. pestis* strains were grown overnight at 28°C with shaking at 250 rpm in 15 ml LB supplemented with appropriate antibiotic. Cultures were vortexed, transferred to 15 ml tubes, and allowed to sit undisturbed at room temperature. 1 ml of culture was removed from the top of the tube at t = 0, 1, 2, and 12 h and the *A*_600_ was determined. To calculate the percentage of sedimentation, the *A*_600_ at different time points was divided by the *A*_600_ of the t = 0 sample.

### Mouse infection assays

*Y. pestis* KIM6+ (pCD1::kan) and KIM6+ Δ*hmsT*Δy3730 (pCD1::kan) strains were grown overnight without shaking in BHI at 28°C and used to inoculate fresh BHI cultures that were incubated overnight in the same conditions. Bacteria were quantified by direct count and serially diluted in PBS. Female 6 to 8-week-old RML Swiss Webster mice were inoculated subcutaneously with 100 CFU of *Y. pestis*, verified by plating the inocula on blood agar. Mice were examined three times daily for two weeks and euthanized upon signs of terminal plague [44]. Plague was verified by culture of *Y. pestis* from triturated suspensions of spleens dissected from euthanized mice. All animal experiments were approved by the Rocky Mountain Laboratories, National Institute of Allergy and Infectious Diseases, National Institutes of Health Animal Care and Use Committee (protocol number 07-44) and were conducted in accordance with all National Institutes of Health guidelines.

## Supporting Information

Figure S1 *Y. pestis* y3730 encodes a c-di-GMP synthesizing diguanylate cyclase (DGC) enzyme whose activity is dependent on the GGDEF domain. A. HPLC profile of a solution of 0.4 nmol synthetic c-di-GMP. B–H. HPLC quantification of intracellular levels of c-di-GMP in the *Y. pestis hmsT* y3730 *hmsP* triple mutant transformed with the empty plasmid vector (B, C) or with the plasmid vector containing *hmsT* (D, E), y3730 (F, G) or the mutated GGAAF allele of y3730 (H). Samples C, E, and G were supplemented with 1 nmol synthetic c-di-GMP. Arrows indicate the c-di-GMP peaks.
(TIF)

Figure S2 Loss of diguanylate cyclase (DGC) activity does not significantly reduce *Y. pestis* virulence in a mouse model of bubonic plague. Incidence of terminal disease in mice after subcutaneous injection of 100 CFU of *Y. pestis* KIM6+ (pCD1-kan) (white boxes) or KIM6+ Δ*hmsT* Δy3730 (pCD1-kan) (black circles) is shown. All ten mice developed terminal plague after injection of the wild type Y. pestis KIM6+, and 9 of 10 mice developed terminal plague after injection of the Δ*hmsT* Δy3730 double mutant.
(TIF)

Table S1 Sequences of primers and probes used in this study.
(DOC)
