# Supplementary figures and images for: Differential Control of Yersinia pestis Biofilm Formation In Vitro and in the Flea Vector by Two c-di-GMP Diguanylate Cyclases
Source: PLoS One. 2011 Apr 29;6(4):e19267. doi: 10.1371/journal.pone.0019267 (PMC3084805; doi:10.1371/journal.pone.0019267)

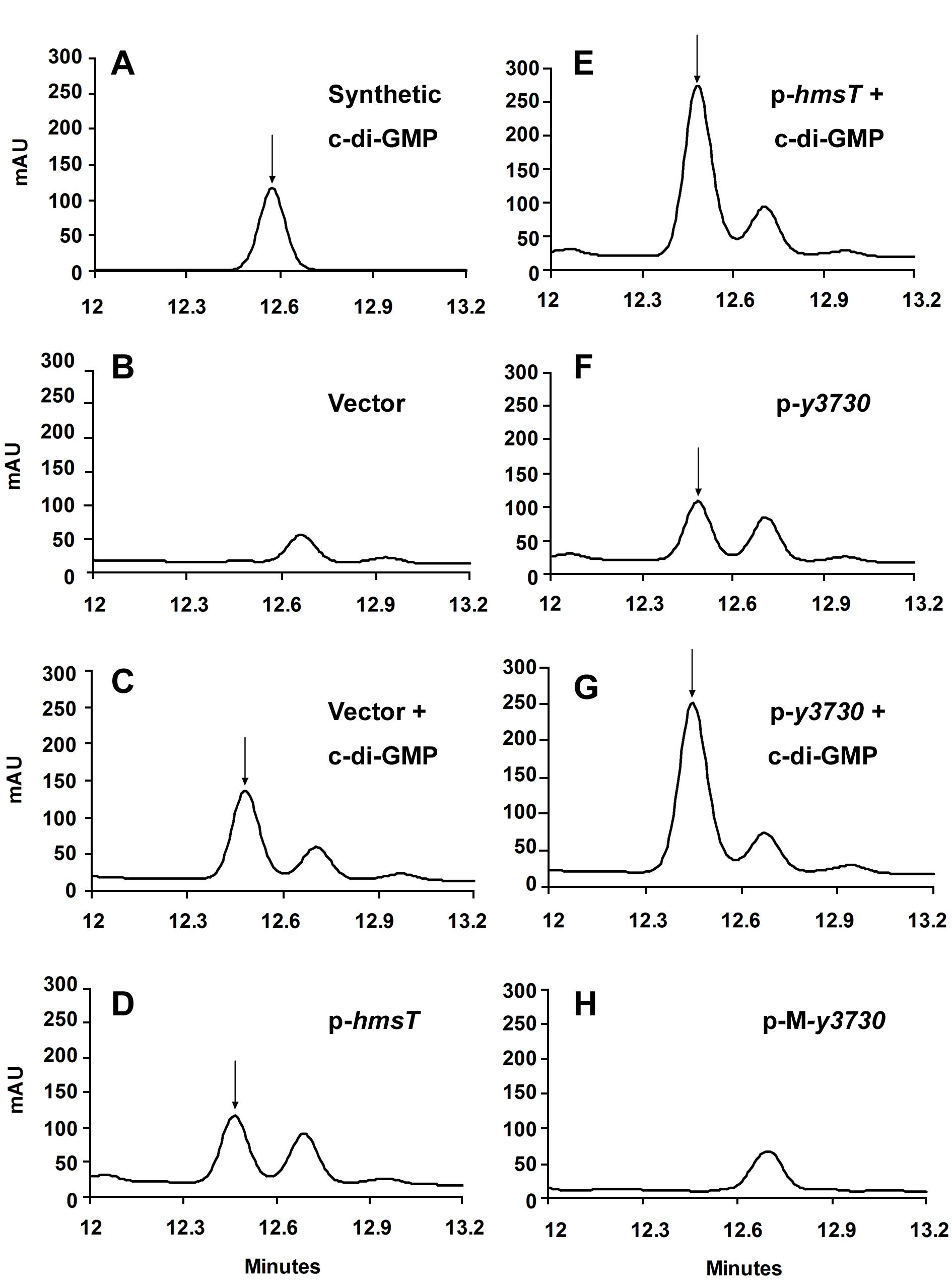

Supplement: Figure S1 — Y. pestis y3730 encodes a c-di-GMP synthesizing diguanylate cyclase (DGC) enzyme whose activity is dependent on the GGDEF domain. A. HPLC profile of a solution of 0.4 nmol synthetic c-di-GMP. B–H. HPLC quantification of intracellular levels of c-di-GMP in the Y. pestis hmsT y3730 hmsP triple mutant transformed with the empty plasmid vector (B, C) or with the plasmid vector containing hmsT (D, E), y3730 (F, G) or the mutated GGAAF allele of y3730 (H). Samples C, E, and G were supplemented with 1 nmol synthetic c-di-GMP. Arrows indicate the c-di-GMP peaks. (TIF) [file pone.0019267.s001.tif]

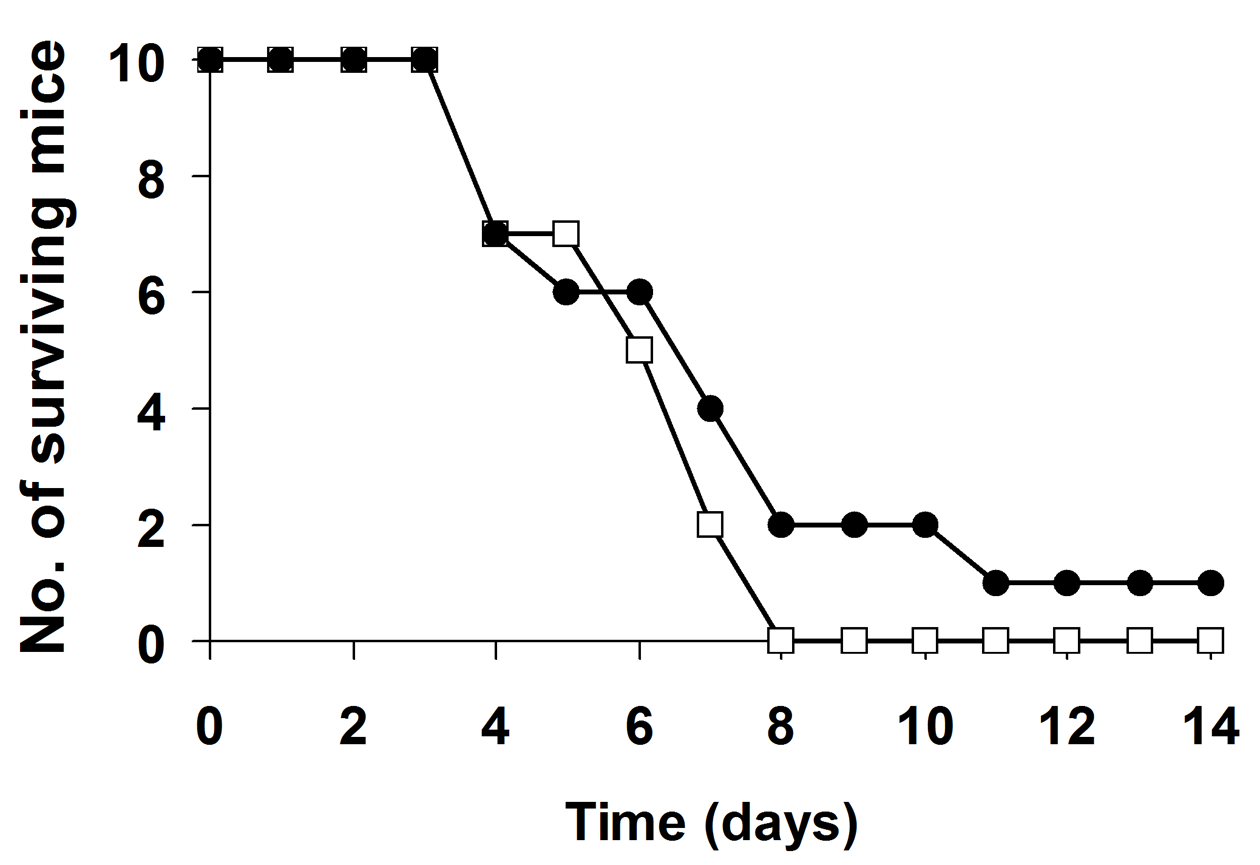

Supplement: Figure S2 — Loss of diguanylate cyclase (DGC) activity does not significantly reduce Y. pestis virulence in a mouse model of bubonic plague. Incidence of terminal disease in mice after subcutaneous injection of 100 CFU of Y. pestis KIM6+ (pCD1-kan) (white boxes) or KIM6+ ΔhmsT Δy3730 (pCD1-kan) (black circles) is shown. All ten mice developed terminal plague after injection of the wild type Y. pestis KIM6+, and 9 of 10 mice developed terminal plague after injection of the ΔhmsT Δy3730 double mutant. (TIF) [file pone.0019267.s002.tif]
