# Supplementary material for: Differential Control of Yersinia pestis Biofilm Formation In Vitro and in the Flea Vector by Two c-di-GMP Diguanylate Cyclases
Source: PLoS One. 2011 Apr 29;6(4):e19267. doi: 10.1371/journal.pone.0019267 (PMC3084805; doi:10.1371/journal.pone.0019267)
Supplement: Table S1 — Sequences of primers and probes used in this study. (DOC) [file pone.0019267.s003.doc]

| Supplementary Table S1. Sequences of primers and probes used in this study | | |
| --- | --- | --- |
| Target gene | Use | Sequence (5’ to 3’) |
| *hmsT* | Deletion | gtctactgacagcacgatattatgcagagtaaattgaatatgaatagccactcctacggtgtaggctggagctgcttcg |
|  |  | caatcattaaaaataataactggcatagagcgtccgtatgttcagtgagttaatcaaggcatatgaatatcctccttag |
|  | TaqMan primers | atcaggctctggtacggatttc |
|  |  | acgcaccacgatatctcttgaac |
|  | TaqMan Probe | cacagcatcgcgaatggccg |
|  |  |  |
| *hmsD* | Cloning | ggaattccgataaaaaaccaatctatgaagccga |
|  |  | cgggatccgttcatcgtgcattagccttct |
|  | Deletion | ccgtaagaaggctaatgcacgatgaacacgataaaaaaccaatctattccggggatccgtcgacc |
|  |  | ctatcctaaactttctgtgttagtcgtattcggctgataggcaaaagtgtaggctggagctgcttcg |
|  | Mutation | gccacctaagcgagccactaaatc |
|  |  | atgatgcgcagcgtttgcggtgttactgaccct |
|  | TaqMan primers | gccttacgggtttatgttgatcac |
|  |  | ggcctcggtggtataactgatg |
|  | TaqMan probe | acctgcatttgatcgcccgcg |
|  |  |  |
| *crr* | TaqMan primers | gccctctggcaataaaatgg |
|  |  | agcatggttggtctcgaaaatt |
|  | TaqMan probe | ctcctgttgacggcaccatcggt |
|  |  |  |
| *hmsP* | Cloning | ggaaaagaccgaaacccac |
|  |  | ggggagcaattaaacccatac |
|  | Deletion | taacaaccaccttattttatgcagtcggaattggcggagaacgagtattccggggatccgtcgacc |
|  |  | ttctcggcgtgttaagtactgctggtggacaccgccatacaaagaggtgtaggctggagctgcttcg |
|  |  |  |
| *y0203* | Cloning | acgatgggtgattcgcttac |
|  |  | aagtgcacaccaacgattca |
|  | Deletion | ggtaatgactgacacattctacgttaatttttgctcaacgatcaggctaacatggccctattacggtgtaggctggagctgcttcg |
|  |  | cccatggatttgagggactctattcatttaattgcactaggcaaggctaagtaatgcgcatatgaatatcctccttag |
|  |  |  |
| *y1612* | Cloning | gtgggccagtaggtcacttg |
|  |  | cgcgatacagcctatcaagg |
|  | Deletion | tcctgctgagatcggataactatcttactgttatctatatacttttccaggtgtaggctggagctgcttcg |
|  |  | gaatacccgcgcgatacagcctatcaaggcgcgctttgccaaaaacattacatatgaatatcctccttag |
|  |  |  |
| *y2472* | Cloning | tgacgcatgcttaagccctcatattcctga |
|  |  | gtacgagctcactccttcctgcaactcgaa |
|  | Deletion | agcgagacagattgataacagactctttgcagcagaatagtgagtgtaggctggagctgcttcg |
|  |  | atgttcagtaactggttcggattaaaaagccgctatcctttgcattccggggatccgtcgacc |
|  |  |  |
| *y2559* | Cloning | tgacgcatgcgtataacctttgccaatgc |
|  |  | ctaagagctctgatcaggatcgaattgctg |
|  | Deletion | tccaagatgtcacccgtgcacgccatgaacagcgggcgctgcaattccggggatccgtcgacc |
|  |  | cggccagcatgtttggccttgtagcaggcaatatcggccaaacgtgtaggctggagctgcttcg |
|  |  |  |
| *y2909* | Cloning | tattgttggcaatctcctgcgaag |
|  |  | ccctatcaccttccactgcg |
|  | Deletion | atgggttcaaacagtgcgttctcccgcaatattttgtccagaattccggggatccgtcgacc |
|  |  | ttaagccagtaagtttttgttactcatcttttcatgacagaagtgtaggctggagctgcttcg |
|  |  |  |
| *y3389* | Cloning | tgacgcatgcttctcggggtcagtcata |
|  |  | ctaagagctcgtaaacgccgatctcctgtc |
|  | Deletion | ccctgatcaccaaccccgctccccaaaccgagccccgaaccgatccccccgctcccccacgtgtaggctggagctgcttcg |
|  |  | acaattaaatgaacaggcaagtgtacaaagccgcttcctgatcgaaaaagcgtagaaaatgcatatgaatatcctccttag |
|  |  |  |
| *y3841* | Cloning | cggtaagtttcccacgttgt |
|  |  | gcaggcaggagaagatgaaa |
|  | Deletion | agcatgacagcgatagcccgccccgccattgctgtttggtaccctgaaacgtgtaggctggagctgcttcg |
|  |  | ggcaggcaggagaagatgaaaggcgatcattatcgccttagggagcgttacatatgaatatcctccttag |
|  |  |  |
| *yadA* | Deletion | atgactaaagattttaagatcagtgtctctgcggcattaatatcgtgtaggctggagctgcttcg |
|  |  | ttaccactcgatattaaatgatgcgttgtacatgacattcgaggacatatgaatatcctccttag |
